# Astrocyte-derived dominance winning reverses chronic stress-induced depressive behaviors

**DOI:** 10.1186/s13041-024-01134-1

**Published:** 2024-08-27

**Authors:** Kyungchul Noh, Junyoung Oh, Woo-Hyun Cho, Minkyu Hwang, Sung Joong Lee

**Affiliations:** 1 Department of Physiology and Neuroscience, Dental Research Institute, Seoul National University School of Dentistry, 1 Gwanak-ro, Gwanak-gu, Seoul, 08826 Republic of Korea; 2 Institute for Neurological Therapeutics Rutgers-Robert Wood Johnson Medical School, Piscataway, NJ 08854 USA; 3 Department of Brain and Cognitive Science, College of Natural Sciences, Seoul National University, Seoul, 08826 Republic of Korea

**Keywords:** Depression, Medial prefrontal cortex, Astrocyte, Dominance behavior, Chronic restraint stress, Winning experience

## Abstract

Individuals with low social status are at heightened risk of major depressive disorder (MDD), and MDD also influences social status. While the interrelationship between MDD and social status is well-defined, the behavioral causality between these two phenotypes remains unexplored. Here, we investigated the behavioral relationships between depressive and dominance behaviors in male mice exposed to chronic restraint stress and the role of medial prefrontal cortex (mPFC) astrocytes in these behaviors. Chronic restraint stress induced both depressive and submissive behaviors. Chemogenetic mPFC astrocyte activation significantly enhanced dominance in chronic stress-induced submissive mice by increasing the persistence of defensive behavior, although it did not affect depressive behaviors. Notably, repetitive winning experiences following mPFC astrocyte stimulation exerted anti-depressive effects in chronic restraint stress-induced depressive mice. These data indicate that mPFC astrocyte-derived winning experience renders anti-depressive effects, and may offer a new strategy for treating depression caused by low status in social hierarchies by targeting mPFC astrocytes.

## Introduction

Individuals with lower social status frequently exhibit comorbid depressive symptoms, underscoring the strong link between social status and mental health [1–3]. Those at the lower end of the social hierarchy are particularly susceptible to depression, which is attributed largely to experiences of social defeat [1–3]. Conversely, individuals with higher social status typically enjoy better self-esteem and overall psychological health [4]. While subordinate behavior may have its advantages, such as reducing the costs associated with excessive social conflict, this involuntary submission is believed to increase the risk of depression and anxiety [5]. Studies have shown that mice subjected to chronic stress exhibit submissive and depressive-like behaviors, which can be mitigated by long-term treatment with anti-depressants like fluoxetine [6]. Moreover, experiences of losing can induce a depressive phenotype in previously dominant mice [7]. However, neurobiological mechanisms underlying the psychological effects of winning experiences remain largely unexplored.

In both humans and animals, past winning experience enhances the chance of future victories [8–10]. Studies have indicated that this so-called winner effect can help to shape social structures and relationships, including the establishment of dominance hierarchies [11, 12]. In addition, the winning experience can influence an individual’s mood states. Research has shown that repeated victories can boost self-confidence and reduce anxiety [13], contributing to higher social status and increased resilience against stress. Conversely, the lack of such experiences can perpetuate feelings of inferiority and depression [14], reinforcing low social status and poor mental health outcomes. Understanding the mechanisms behind these phenomena can offer insights into therapeutic approaches for mood disorders and social dysfunctions, particularly in the context of hierarchical social settings.

We have previously reported that astrocytes in the medial prefrontal cortex (mPFC) are key brain substrates controlling mouse dominance behaviors [15]. Optogenetic and chemogenetic activation of mPFC astrocytes can elevate the social rank of previously subordinate mice by altering the balance between excitatory and inhibitory neuronal activity (E/I balance) in the region. Furthermore, reductions in astrocyte activity in the mPFC have been linked to chronic stress-induced depressive behavior in mice [16]. Additionally, reduced astrocyte density in the mPFC is a clinical marker in the post-mortem brains of patients with major depressive disorder (MDD) [17–21]. Given these findings, astrocytes in the mPFC likely play a role in both dominance and depressive behaviors.

In our study, mice subjected to chronic restraint stress displayed both depressive-like behaviors and reduced dominance. By chemogenetically activating astrocytes in the mPFC, we enabled these stressed mice to achieve dominance in tube tests. Interestingly, such winning experiences in tube tests exerted significant anti-depressant effects in the stressed mice. This behavioral study suggests that the physiological effects of the winning experience in competition can influence affective states, such as depression.

## Results

### Chronic restraint-stressed mice show enhanced depressive and reduced dominance behaviors

**Fig. 1:**
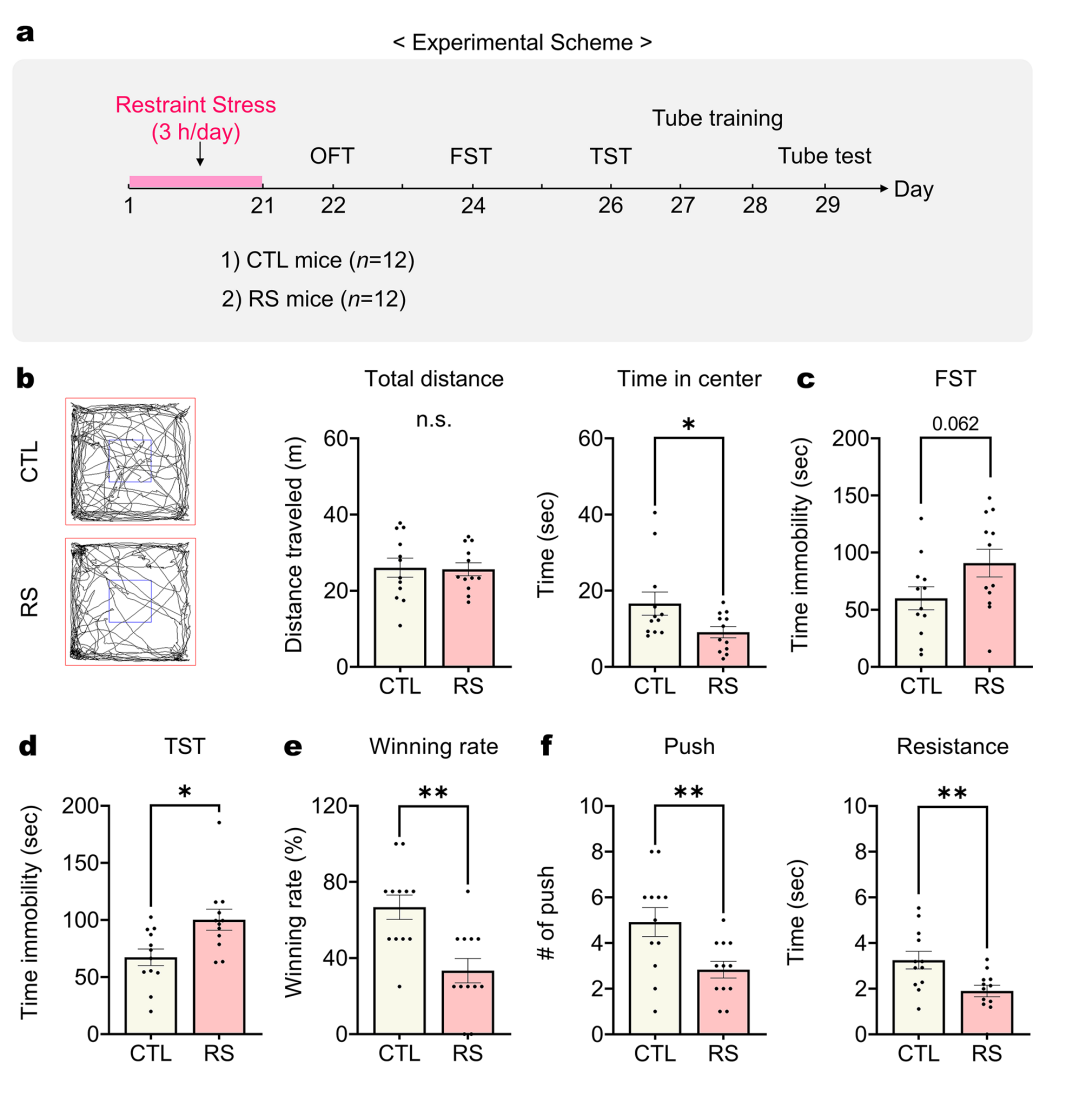
Chronic restraint stress simultaneously alters both depressive and dominance behaviors. (**a**) Experimental scheme outlining the procedure for chronic restraint stress and behavior tests for control (CTL) and restraint stress (RS) mice. (**b**) Total distance and time spent in the center during the open field test (OFT) (*n* = 12 mice per group). Student’s *t*-test. (**c** and **d**) Time immobility measured in the forced swim test (FST) and tail suspension test (TST) (*n* = 12 mice per group). Student’s *t*-test. (**e**) Winning probability in the tube test (*n* = 12 mice per group). Student’s *t*-test. (**f**) Number of pushes and time resistance shown by experimental mice during the tube test (*n* = 12 mice per group). Student’s *t*-test. *: *p* < 0.01; **: *p* < 0.005; n.s.: not significant. Data are presented as mean ± s.e.m

To investigate the impact of chronic restraint stress on behavioral deficits in mice, we subjected mice to restraint stress for three weeks. Subsequently, we conducted behavioral tests to assess depressive and dominance behaviors (Fig. 1a). The findings revealed that mice exposed to restraint stress (RS mice) displayed depressive-like behaviors. They spent less time in the center during the open field test (OFT) (Fig. 1b), and showed increased immobility time during the forced swim test (FST) and tail suspension test (TST) (Fig. 1c and d). Moreover, following two days of tube training, RS mice displayed a lower winning rate in the tube test compared to their control counterparts (CTL mice), showing reduced push and resistance behaviors (Fig. 1e and f). These results indicate that chronic restraint stress leads to concurrent depressive-like and reduced dominance (submissive) behaviors.

### Acute chemogenetic stimulation of prefrontal astrocytes mitigates submissive behavior but not depressive behavior

**Fig. 2:**
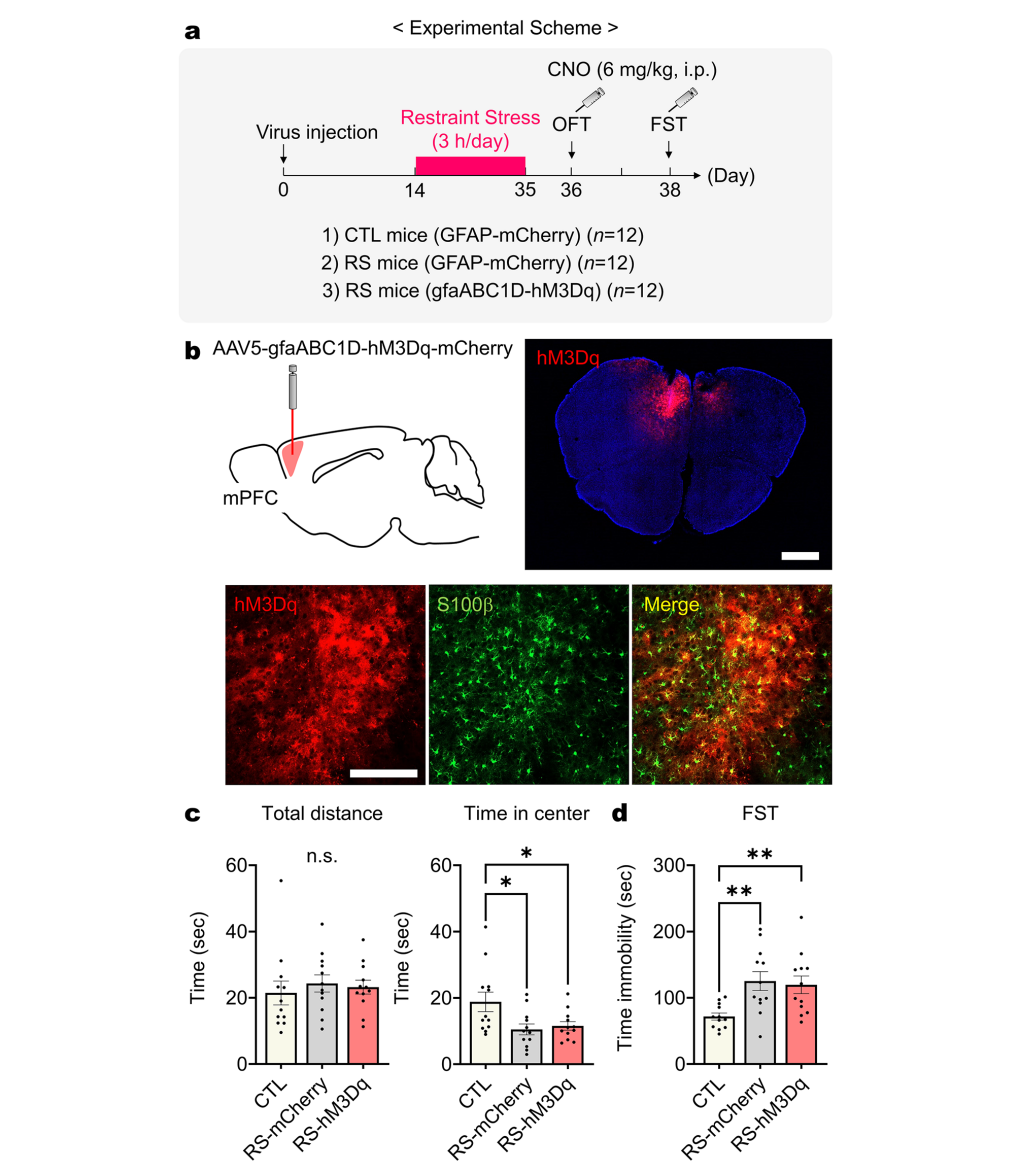
Depressive behaviors induced by chronic restraint stress are not reversed by acute chemogenetic mPFC astrocyte stimulation. (**a**) Experimental scheme to measure depressive behaviors with acute chemogenetic mPFC astrocyte stimulation in RS mice. (**b**) Illustration of viral delivery to mPFC astrocytes and verification of astrocyte-specific hM3Dq expression in the mPFC region following AAV5-gfaABC1D-hM3Dq-mCherry virus injection. Scale bar: 1 mm and 200 μm. (**c**) Total distance and time spent in the center during the OFT (*n* = 12 mice per group). One-way ANOVA, Bonferroni *post-hoc* analysis. (**d**) Time immobility measured in the FST (*n* = 12 mice per group). One-way ANOVA, Bonferroni *post-hoc* analysis. *: *p* < 0.01; **: *p* < 0.005; n.s.: not significant. Data are presented as mean ± s.e.m

Previous studies have highlighted the critical role of prefrontal astrocytes in managing and regulating depression [16] and dominance behaviors [15]. Building on these findings, we explored whether activating astrocytes in the medial prefrontal cortex (mPFC) could mitigate chronic stress-induced depressive and submissive behaviors. To investigate this, we utilized chemogenetic stimulation of astrocytes in the mPFC. Specifically, we bilaterally administered AAV5-gfaABC1D-hM3Dq-mCherry into the prelimbic (PL) region of the mPFC in wild-type male mice (Fig. 2a). Following a two-week period post-viral injection, these virus-injected mice were subjected to restraint stress for three consecutive weeks. Behavioral assessments were conducted using the OFT and FST, accompanied by intraperitoneal injections of clozapine N-oxide (CNO) (6 mg/kg). Expression of hM3Dq was specific to astrocytes (S100β-positive cell) within the PL region of the mPFC (Fig. 2b). Despite the chemogenetic stimulation of mPFC astrocytes, the CNO-injected hM3Dq-expressing RS mice still exhibited decreased time spent in the center during the OFT and increased immobility time in the FST (Fig. 2c and d) indicating that mPFC astrocyte activation did not per se rescue mice from the chronic stress-induced depressive behaviors.

**Fig. 3:**
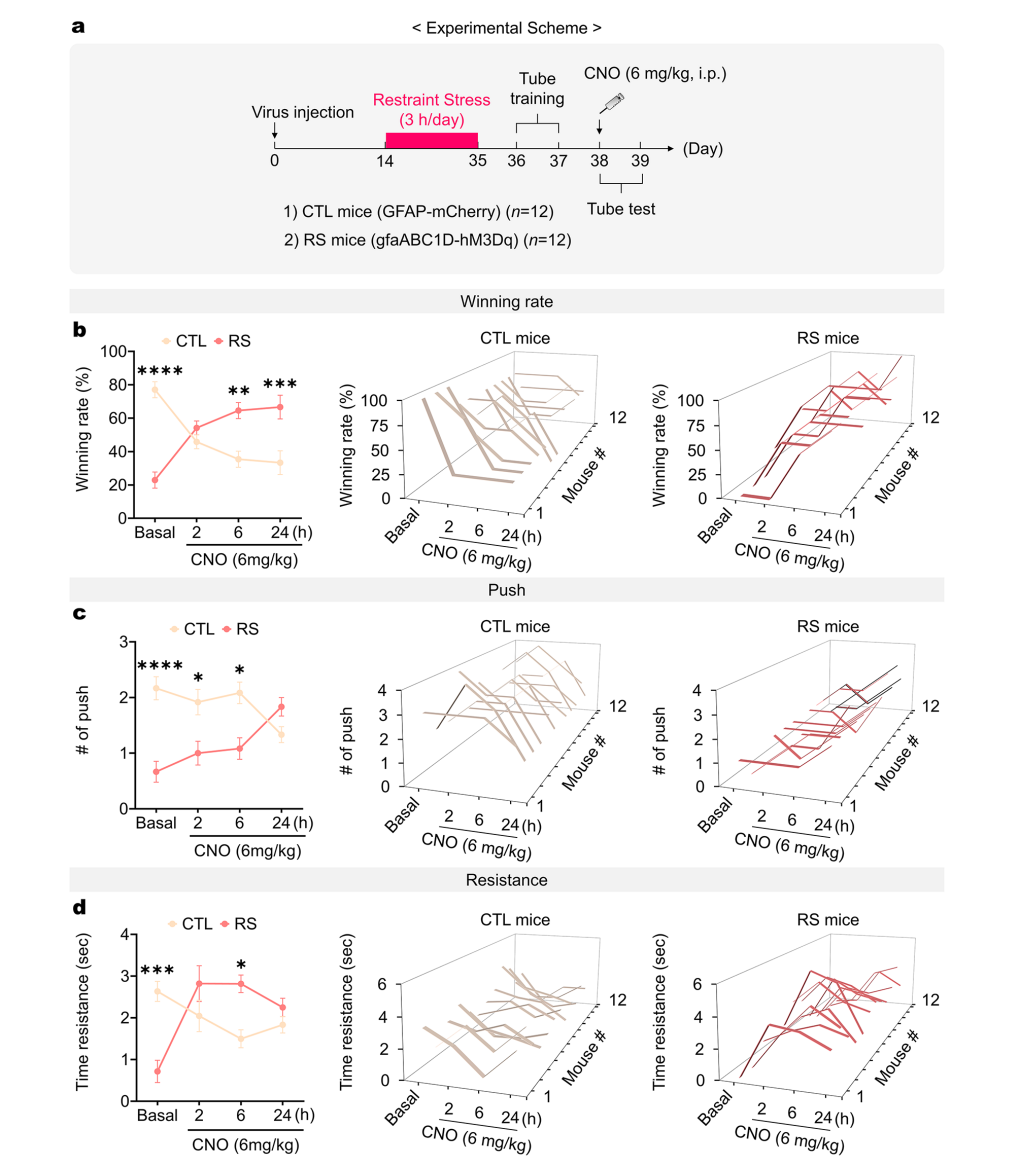
Acute chemogenetic mPFC astrocyte stimulation increases dominance winning of RS mice during tube competition. (**a**) Experimental scheme to measure dominance behaviors with acute chemogenetic mPFC astrocyte stimulation in RS mice. Dominance behavioral changes in mPFC astrocyte-stimulated RS mice: (**b**) winning rate, (**c**) number of pushes, and (**d**) time of resistance (*n* = 12 mice per group). Student’s *t*-test. *: *p* < 0.01; **: *p* < 0.005; ***: *p* < 0.001; ****: *p* < 0.0001. Data are presented as mean ± s.e.m

Next, we conducted a tube test to evaluate the influence of mPFC astrocytes on dominance behaviors in RS mice. After three weeks of restraint stress, we performed tube training for two consecutive days (Fig. 3a). Following tube training, we administered a baseline tube test against CTL mice. Subsequently, CNO was injected into RS mice (6 mg/kg, i.p.), and the tube test was repeated 2, 6, and 24 h post-injection. Initially, RS mice demonstrated a significantly lower winning rate at baseline (Fig. 3b). However, they achieved a winning rate comparable to that of non-RS mice 2 h after CNO injection. Remarkably, at both 6 and 24 h post-CNO injection, RS mice exhibited a significantly higher winning rate than matched controls (CTL mice) (Fig. 3b). Furthermore, CNO-injected RS mice showed a significant increase in pushing behavior 24 h after the CNO injection (Fig. 3c). Additionally, RS mice exhibited a significant alteration in resistance behavior starting from 2 h post-CNO injection, which lasted up to 24 h post-injection (Fig. 3d). These findings suggest that chemogenetically mPFC astrocyte-stimulated RS mice initially enhance their resistance behavior to achieve winning and subsequently engage in pushing behaviors to maintain dominance over their opponents.

### Winning experience mitigates chronic stress-induced depressive-like behaviors

**Fig. 4:**
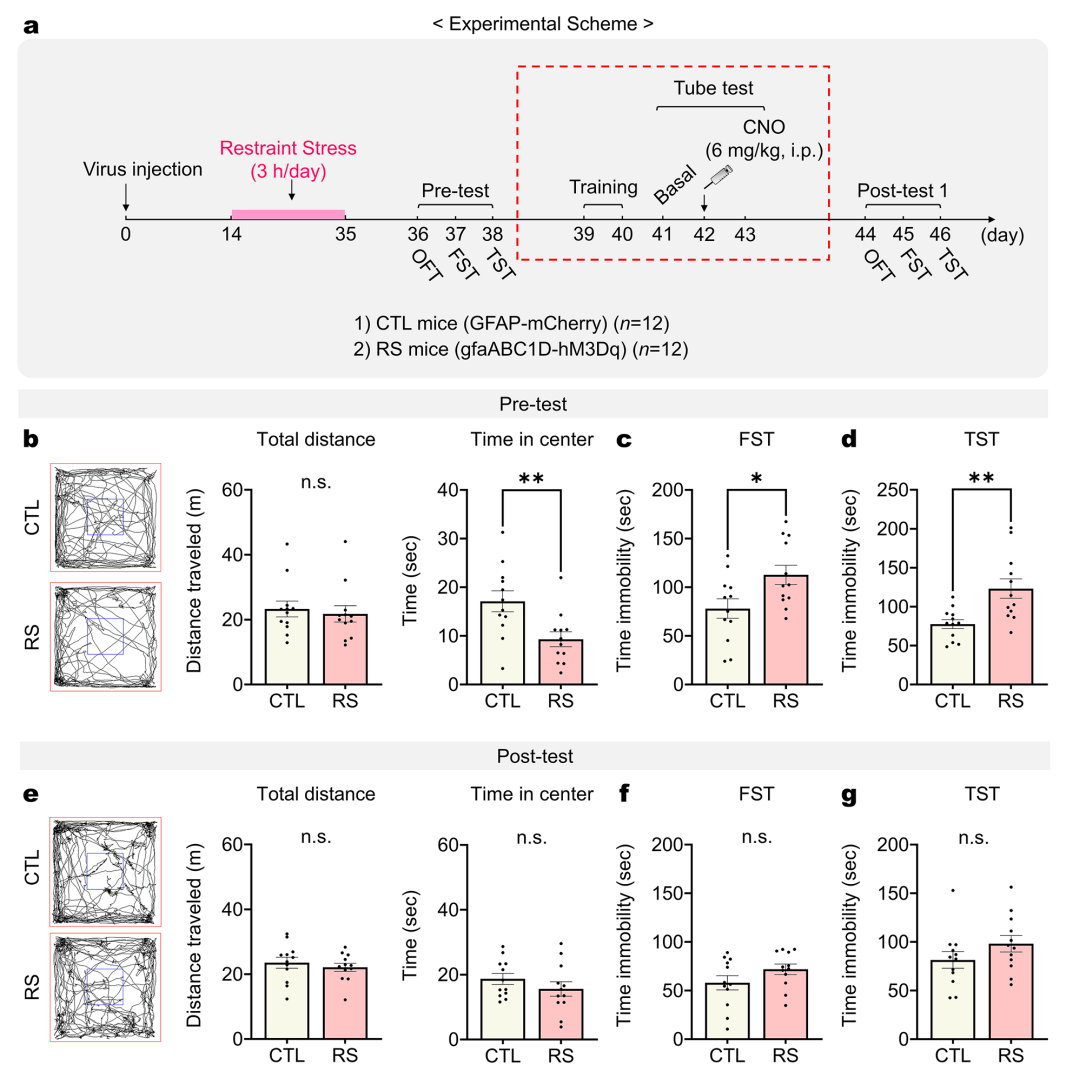
Experience of dominance winning reverses chronic stress-induced depressive behaviors. (**a**) Experimental scheme for depressive behavior tests before and after the tube test. The dashed red square indicates the procedure for tube training and tube testing with chemogenetic stimulation of mPFC astrocytes of RS mice. Pre-test: (**b**) Total distance and time spent in the center during the OFT, (**c** and **d**) time immobility measured in the FST (**c**) and TST (**d**) between CTL and RS mice (*n* = 12 mice per group). Student’s *t*-test. Post-test: (**e**) Total distance and time spent in the center during the OFT, (**f** and **g**) time immobility measured in the FST (**f**) and TST (**g**) between CTL and RS mice (*n* = 12 mice per group). Student’s *t*-test. *: *p* < 0.01; **: *p* < 0.005; n.s.: not significant. Data are presented as mean ± s.e.m

**Fig. 5:**
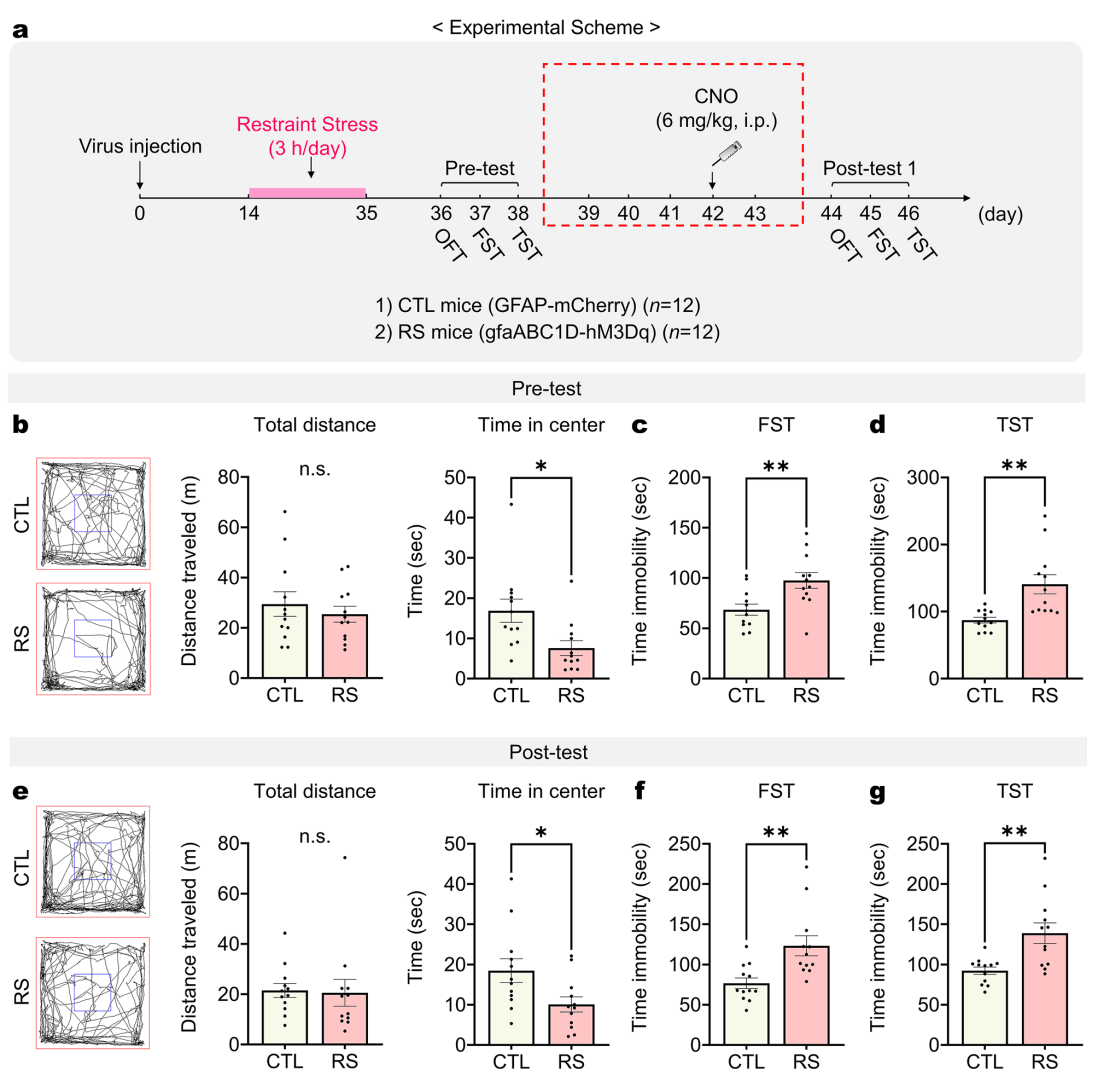
Chronic stress-induced depressive behaviors are not reversed without the experience of winning. (**a**) Experimental scheme for depressive behavior tests without the tube test. The dashed red square indicates the procedure for chemogenetic stimulation of mPFC astrocytes in RS mice. Pre-test: (**b**) Total distance and time spent in the center during the OFT, (**c** and **d**) time immobility measured in the FST (**c**) and TST (**d**) between CTL and RS mice (*n* = 12 mice per group). Student’s *t*-test. Post-test: (**e**) Total distance and time spent in the center during the OFT, (**f** and **g**) time immobility measured in the FST (**f**) and TST (**g**) between CTL and RS mice (*n* = 12 mice per group). Student’s *t*-test. *: *p* < 0.01; **: *p* < 0.005; n.s.: not significant. Data are presented as mean ± s.e.m

Depression and lack of dominance are interrelated, with their comorbidity significantly impacting quality of life [1, 2]. Despite the lack of recovery in stress-induced depression by chemogenetic mPFC astrocyte stimulation, it was hypothesized that experiences of dominance, such as winning in tube tests, could positively influence affective disorders such as depression. To explore the idea, we aimed to provide RS mice with winning experiences through the tube test, and then assessed changes in depressive behaviors before and after these winning experiences (Fig. 4a). Following the injection of hM3Dq and a three-week period of restraint stress, we conducted baseline behavioral assessments using OFT, FST and TST (pre-test). Next, 2 days after tube training, we performed tube testing with chemogenetic mPFC astrocyte stimulation and then conducted the OFT, FST, and TST again (post-test). Initially, RS mice demonstrated behaviors indicative of depression, such as reduced time spent in the center during the OFT and increased immobility time in both the FST and TST, compared to CTL mice, as already illustrated in Fig. 1. However, following their winning in the tube test, these RS mice exhibited improvements in depressive-like behaviors. RS mice showed comparable distance traveled and time spent in the center of the OFT during post-test (Fig. 4e). Additionally, their immobility times in the FST and TST became comparable to those of CTL mice (Fig. 4f and g). To determine whether such acute anti-depressant effects observed were attributable to chemogenetic stimulation of mPFC astrocytes rather than the experience of winning, we replicated the experiments without conducting the tube test (Fig. 5a). In this experimental setup, we still administered CNO (6 mg/kg, i.p.). The results indicated that, in the absence of winning experiences through the tube tests, RS mice maintained significant depressive-like behaviors despite the stimulation of mPFC astrocytes with CNO (Post-test; Fig. 5e-g). Collectively, these findings suggest that the mitigation of some depressive-like behaviors in RS mice may stem from winning experiences in the tube test, rather than from a physiological recovery facilitated by mPFC astrocyte stimulation.

### K-means clustering analysis confirms the anti-depressant effects of mPFC astrocyte-derived winning experiences

**Fig. 6:**
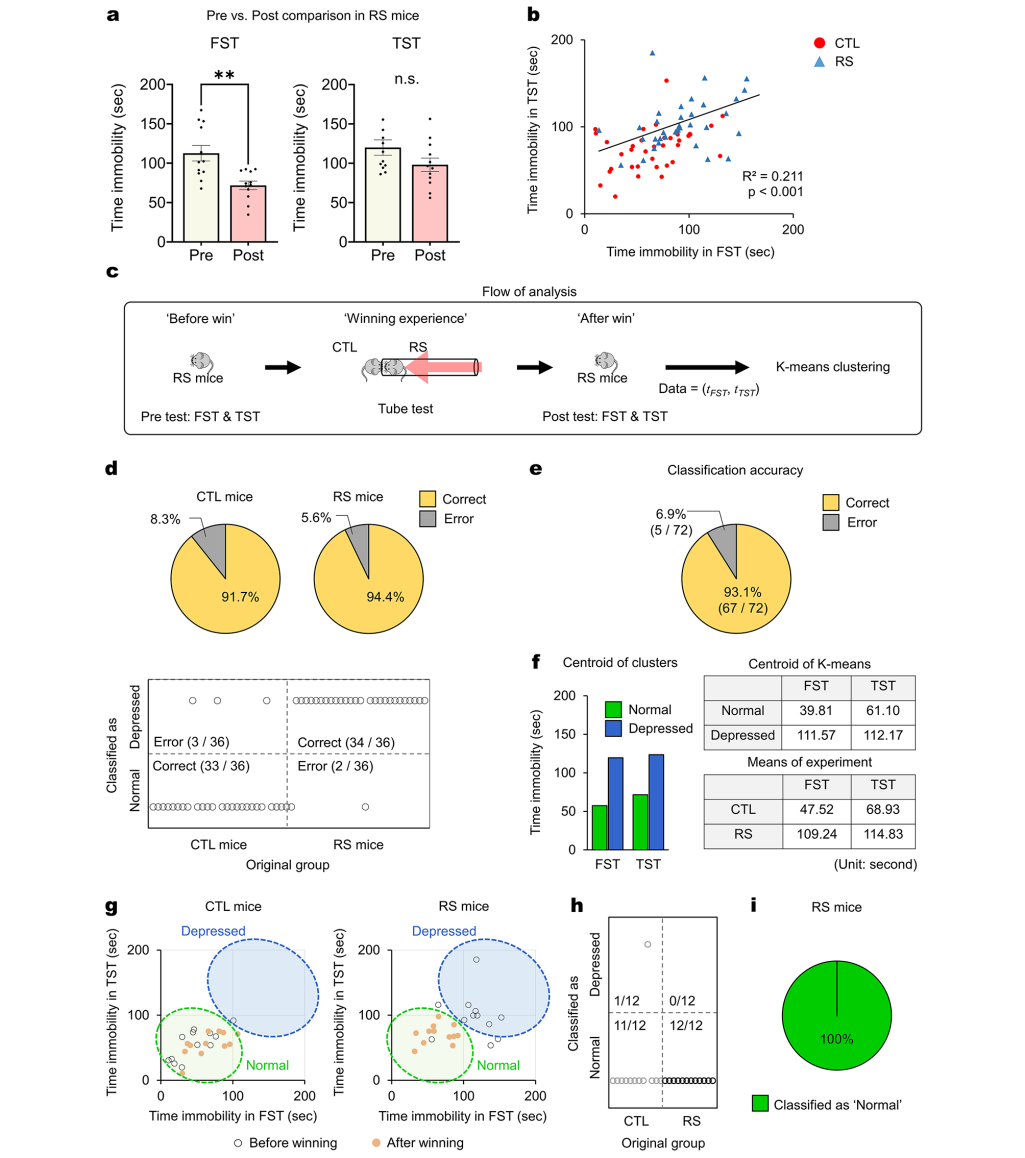
Behavior of RS mice after repetitive winning is classified as CTL-like behavior by K-means clustering analysis. (**a**) In RS mice, the immobility times in the FST and TST after tube tests (Post-test) markedly decreased compared with those of Pre-tests, although the *p* values were higher than 0.05. Paired *t*-test. (**b**) Pearson’s correlation analysis indicates a significant positive correlation between the immobility values in both the ‘Pre-test’ and ‘Post-test’ phases of the FST (*n* = 36) and TST (*n* = 36) (R^2^ = 0.211; *p* < 0.001). (**c**) Scheme of K-means clustering analysis. RS mice, which exhibit both submissive and depressive phenotypes, were subjected to repeated tube tests with mPFC astrocyte stimulation, allowing winning experiences. The immobility times in the FST and TST before (Pre-test) and after (Post-test) the tube tests were collected and analyzed by K-means clustering analysis. (**d**) The data were clustered into two groups: Normal and Depressed. The immobility time values from Pre-tests (FST and TST) of CTL and RS mice were used to classify the data into two groups. (*n* = 36). Among 36 CTL data, 33 were classified as Normal, and 3 were classified as Depressed (Classification accuracy = 91.7%). Among 36 RS data, 34 were classified as Depressed and 2 as Normal (classification accuracy = 94.4%). (**e**) Combining both CTL and RS mice, the K-means clustering method classified the behavior data with 93.1% accuracy. Only 5 of 72 data points were incorrectly classified (error rate = 6.9%). (**f**) Centroids of each classified cluster were similar to the mean values of immobility measured from experiments. (**g**) Individual scatter plots of immobility times of CTL and RS mice measured from the FST and TST after tube tests. These scatter plots clearly show that immobility times from the FST and TST of astrocyte-stimulated RS mice are classified in the Normal cluster. (**h** and **i**) Results of K-means clustering of RS mice that had winning experiences after mPFC astrocyte stimulation. In astrocyte-stimulated RS mice, all 12 mice were classified as Normal (100%; 12 out of 12). **: *p* < 0.005; n.s.: not significant. Data are presented as mean ± s.e.m

While our observations indicated comparable levels of immobility during the FST and TST between CTL mice and mPFC astrocyte-stimulated RS mice with winning experiences, as highlighted in Fig. 4 (Fig. 4e-g), a direct comparison of immobility times between the ‘Pre-test’ and ‘Post-test’ phases for RS mice did not yield statistical significance in the TST (Fig. 6a). Therefore, to quantitatively assess the development of depressive-like phenotypes in RS mice post-winning, we employed K-means clustering analysis, a mathematical analysis to categorize complex datasets into distinct subgroups [22, 23]. By plotting all immobility values obtained from this study, we identified a significant positive correlation between time spent immobile in the FST and TST (R^2^ = 0.211; *p* < 0.001) (Fig. 6b). The data clearly indicated that mice displaying high immobility in the FST also showed high immobility in the TST (Fig. 6b). Through K-means clustering analysis (Fig. 6c), we classified these values into two groups: Normal and Depressed. Of the two groups, the algorithm accurately classified 91.7% and 94.4%, respectively (Fig. 6d). Misclassification was minimal, with only three out of 36 CTL data and two out of all RS data being incorrectly identified as Depressed and Normal, respectively (Fig. 6d). When combining data from both CTL and RS mice, the overall classification accuracy stood at 93.1% (Fig. 6e). The centroids of each group (Normal and Depressed) closely aligned with the mean immobility times obtained from experiments on CTL and RS mice, respectively (Fig. 6f).

Further analysis of the Post-test immobility data for RS mice who had winning experiences following mPFC astrocyte stimulation revealed that all were classified as Normal (Fig. 6g-i). These findings strongly support the acute anti-depressant effect of winning experiences facilitated by mPFC astrocyte stimulation and underscore the efficacy of targeting mPFC astrocytes through the tube test to achieve acute anti-depressant outcomes.

## Discussion

A causal relationship between low social status and depression has been proposed in clinical studies [1, 2], yet it had not been investigated in animals. In this study, we adopted a RS mouse model that exhibited both depressive and submissive behaviors to explore the potent link between these two behaviors. First, we tested whether chemogenetic stimulation of mPFC astrocytes induces dominant behavior in RS-induced depressive mice. Similar to unstressed normal mice [15], mPFC astrocyte stimulation increased resistance behavior in RS mice during the tube test. Notably, with repeated tube competition, chemogenetic activation of mPFC astrocytes in RS mice caused a significant behavioral shift from defensive resisting to offensive pushing, resulting in reversal of their final winning odds against control mice within a day. These data suggest that mPFC astrocyte stimulation exerts potent dominance-enhancing effects in stress-induced depressive mice.

The most important finding of our study is that the experience of winning, facilitated by mPFC astrocyte stimulation, induced anti-depressive effects in RS mice. Notably, these anti-depressive effects from mPFC astrocyte stimulation were not observed without winning experience in the tube test. This indicates that mPFC astrocyte activation alone is not sufficient; rather, winning experiences in the tube competition are required for the anti-depressive effects. It has been proposed that repetitive winning experiences result in ‘winner effect’ [24, 25]. Although speculative, it can be conceived that repeated tube test winning experiences, aided by mPFC astrocyte stimulation, helped form a ‘winning memory’ in the RS mice, altering their depressive behavior.

Depression is associated with changes in the processing and representation of reward, linked to anhedonia, one of the core symptoms of the disorder [26–29]. Numerous neuroimaging studies have reported that, compared to healthy controls, individuals with MDD exhibit abnormal activities in brain regions related with reward processing, such as striatum [26, 30] and nucleus accumbens [31–33] in response to rewards. These findings suggest that dysregulated reward processing may underlie the reward-processing abnormalities observed in MDD, providing an empirical foundation for a more refined understanding of the reward circuitry in MDD. According to these studies, the experience of winning as induced by mPFC astrocyte stimulation may have acutely triggered the reward processing in chronic stress-induced depressive mice, and this temporary reward effect might have led to the observed anti-depressive behaviors in RS mice. A prior study indicated that prefrontal astrocytes increase their calcium response when induced by dopamine via dopamine D1 or D2 receptors in adult mice [34]. Moreover, fast calcium signals in mPFC astrocytes are correlated with dopamine release and are mediated by α1-adrenergic receptors, which regulate mouse locomotion [35]. According to these studies, dopamine may be released immediately after dominance winning, triggering a calcium response in mPFC astrocytes. This response may stimulate the dopaminergic reward circuitry through dopamine or adrenergic receptors. Therefore, understanding the underlying mechanisms that sustain reward processing controlled by prefrontal astrocytes could provide insights into developing more effective treatments for stress-induced depression.

Self-awareness of submissiveness might be a key factor influencing depressive mood in subordinate subjects, who are often addressed by cognitive behavior-based therapy (CBT) for depression [36, 37]. Our findings on the unexpected roles of mPFC astrocytes in promoting dominance and inducing rapid anti-depressive behaviors might help elucidate the biological basis of CBT for depression. Additionally, the rapid onset of anti-depressive behavior within a day of astrocyte-stimulated tube test winning is remarkable. This is especially significant considering that traditional anti-depressant drugs, such as SSRIs, require several weeks or even months to exert their effects [38, 39]. Thus, astrocyte activation-induced behavioral changes may offer a more efficient strategy for depression treatment. Although the precise cellular and molecular mechanisms underlying the anti-depressive effects of astrocyte-stimulated winning experiences remain unclear, our results could have clinical implications for treating depression associated with low social status by targeting mPFC astrocytes.

## Methods

### Animals

Six- to 16-week-old male C57BL/6J mice of similar body weights were used for all experiments. The animals were housed and maintained in a controlled environment at 22–24 °C and 55% humidity with 12-h light/dark cycles and fed regular rodent chow and tap water ad libitum. Animal experiments were approved by the Institutional Animal Care and Use Committee (IACUC) of Seoul National University (#SNU-220701-1-2).

### Stereotaxic viral delivery

Nine- to 10-week-old wild-type male mice that showed stable hierarchies in their cages were used for viral delivery. The animals were anesthetized with isoflurane and secured in a stereotaxic frame (Stoelting Co., Wood Dale, IL, USA). Holes the size of the injection needle were drilled into the skull, and bilateral injection with 0.7 μl (titer 10^13^ GC/ml) of adeno-associated virus, serotype DJ (AAVDJ) and 5 (AAV5), were administered on each side. The injection syringe (Hamilton Company, Reno, NV, USA) delivered the AAVDJ and AAV5 at a constant volume of 0.1 μl/min using a syringe pump (Stoelting Co.). The needle was left in place for 3 min after each injection to minimize the upward flow of the viral solution after raising the needle. The injection coordinates of the prelimbic (PL) cortex of the medial prefrontal cortex (mPFC) were AP: +2.43, ML: ±0.20, DV: -1.80 from bregma. AAVDJ-GFAP-mCherry was purchased from the Korea Institute of Science and Technology (Seoul, Korea), and pXac2.1-gfaABC1D-hM3Dq-mCherry plasmid was purchased from Addgene (#92284; Watertown, MA, USA) and packaged in AAV5 (AAV5-gfaABC1D-hM3Dq-mCherry) by UPenn Vector Core (Philadelphia, PA, USA). Animals injected with virus were used for behavioral assessment 3–4 weeks post-injection. Animals injected with AAVDJ or AAV5 but showing no detectable viral expression in the target region were excluded from analyses.

### Chronic restraint stress

Mice were individually placed into 50-ml polypropylene conical tubes with a nose-hole for ventilation (3 h/day) for 21 consecutive days. After restraint, the mice were returned to their home cages. One day after the last restraint session, the mice were weighed and used for further experiments. The mice that underwent chronic restraint stress were designated RS mice, and the unstressed control mice were designated control mice (CTL mice).

### Behavioral studies

On all behavioral testing days, animals were moved to the test room and left to habituate there for at least 1 h. The light conditions in the test room were maintained at the same intensity (100 lx) as the animal rooms under daylight conditions.

### Tube test

The tube test was conducted as previously described. We used a transparent Plexiglas tube 30 cm long with a 3-cm inner diameter. Six-week-old male mice were housed in groups of 4 for at least 2 weeks before the tube test. Before the main test, each mouse was trained to go through the tube for 10 trials over two consecutive days. On the test day, pairs of mice were released into the opposite ends and met in the middle of the tube. The tube test was performed for 2 min. If no winner or loser was decided within 2 min, the test was repeated. Between trials, the tube was cleaned with 70% ethanol. Within each cage, the four mice were randomly assigned such that each mouse would meet every other mouse in the group only once, resulting in six matches per cage. All 6 pairs of mice were tested daily in a round robin design, and the ranks were determined by the total number of wins. Only cage mates who maintained stable ranks for 3 or 4 days were used for further experiments. The behaviors were videotaped, and both pushing and resistance behaviors were counted. The test mouse was matched 4 times against mice from the other group. The winning rate of each test mouse was calculated by counting its total number of wins.

### Tube test with chemogenetic mPFC astrocyte stimulation

We injected AAV5-gfaABC1D-hM3Dq-mCherry. Clozapine *N*-oxide (CNO) (4936; Tocris, Minneapolis, MN, USA) was dissolved and diluted in normal saline solution with 0.5% DMSO (6 mg/kg). After 3 weeks of virus injection, we performed 2 days of tube training and basal tube test for 3 consecutive days without CNO injection. The following day, CNO was intraperitoneally injected into test mice. Then, we performed the tube test 2, 6, and 24 h after injection.

### Open field test (OFT)

The apparatus consisted of a brightly illuminated 40 cm x 40 cm square arena surrounded by a wall 40 cm high. Mice were individually placed in the center of the arena, and their locomotion activity was monitored by an automatic system for 10 min. Total distance and time spent in the center zone per minute were analyzed by an automated video tracking system (SMART; Panlab SL, Barcelona, Spain). The activity chamber was cleaned with 70% ethanol after each use to eliminate any olfactory cues from the previously tested mouse.

### Elevated plus maze (EPM)

The behavioral apparatus consisted of two open arms (width 5 cm x length 30 cm) and two closed arms (width 5 cm x length 30 cm) elevated 60 cm above the floor and dimly illuminated. Mice were placed individually in the center of the maze facing an open arm and allowed to freely explore for 5 min. The time spent in each arm was analyzed using a video tracking system. The maze was cleaned with 70% ethanol after each test to prevent olfactory influence from the previously tested mouse.

### Forced swim test (FST) and tail suspension test (TST)

We conducted the FST by placing each animal individually in a transparent cylinder filled with water (23–25 °C; depth 15 cm) for 5 min. Immobility in the FST was defined as a state in which the mouse made only the movements necessary to keep its head above the surface. The TST was performed by hanging each animal from the top of a square box for 5 min. Both trials were videotaped and immobility time was analyzed using a video tracking system.

The experimental protocols for the OFT, EPM, FST, and TST were designed to take into account how potential stress from a previous test could affect mouse behavior in other tests. The behavioral assessments were performed in the following sequence to minimize such stress effects: OFT, EPM, FST, and TST.

### Immunohistochemistry

Whole brains were saline-perfused, fixed in 4% paraformaldehyde in 0.1 M PBS overnight at 4 °C, and dehydrated with 30% sucrose for 3 days. Coronal sections with a thickness of 50 μm were incubated in cryopreservation solution at -20 °C until immunohistochemical staining was performed. The sections were blocked in a blocking solution containing 5% normal donkey serum (Jackson Immunoresearch, Bar Harbor, ME, USA), 2% BSA (A7638, Sigma-Aldrich, St. Louis, MO, USA), and 0.1% Triton X-100 (0694, VWR Life Science, Solon, OH, USA) for 1 h at room temperature. The sections were then incubated with rabbit S100β (ab52642, 1:500; Abcam) antibodies overnight at 4 °C in blocking solution. After being washed with 0.1 M PBS containing 0.1% Triton X-100, the sections were incubated for 2 h with FITC-conjugated secondary antibodies (711-095-152, 1:200; Jackson Immunoresearch) in blocking solution at room temperature, washed 3 times, and then mounted on gelatin-coated slide glass using Vectashield (Vector Laboratories, Inc., Burlingame, CA, USA). Fluorescent images of the mounted sections were obtained with a confocal microscope (LSM800; Carl Zeiss, Jena, Germany).

### Pearson’s correlation analysis and K-means clustering

K-means clustering analysis is one of the simplest and most popular unsupervised machine learning algorithms. It aims to separate scattered data into *k* clusters in which each data point belongs to the cluster with the nearest mean (centroid). This method has been successfully employed in various biological fields to identify and classify complex biological features [40–43].

We plotted individual mouse immobility values into a 2-dimensional space by defining *M*_*n*_ (*t*^FST^, *t*^TST^), in which *M* means mouse and *n* indicates the mouse number. We first performed a Pearson’s correlation analysis to verify whether the immobility values in the FST and TST correlated with each other. The FST immobility values of 72 data, including all CTL (*n* = 36) and RS (*n* = 36), showed a significant positive correlation with the TST immobility values. We performed a K-means classification analysis to divide the immobility values into two groups (*k* = 2). Classification was started from two random points for every cluster, and we performed 1000 iterative calculations to optimize the positions of the centroids in each cluster. After 1000 iterations, the centroids of each classified cluster were determined, and we compared the centroids with the values measured from experiments. Centroids of each classified group were similar to experimental values, and we then verified classification accuracy. The data were classified into ‘Normal’ and ‘Depressed’ groups with 93.1% classification accuracy. Among the 36 CTL data, 33 were correctly classified as ‘Normal’ and 3 were incorrectly classified as ‘Depressed’ (91.7% classification accuracy). Among the 36 RS data, 34 were correctly classified as ‘Depressed’ and 2 mice were incorrectly classified as ‘Normal’ (94.4% classification accuracy). Next, we performed K-means classification using the FST and TST immobility values obtained from the non-RS and RS mice after tube testing. The *k* value was again 2, and the classification iteration was 1000. Both Pearson’s correlation and K-means classification analyses were performed in MATLAB (R2019a).

### Statistics

Statistical significance was determined using the two-tailed Student’s *t*-test and paired *t*-test for comparisons between two groups. For multiple comparisons, one-way analysis of variance (ANOVA) with Bonferroni’s multiple comparison tests was used. All data are presented as mean ± s.e.m., and differences were considered statistically significant when the *p*-value was less than 0.01.

## Data Availability

All data generated or analyzed during this study are included in this published article.

## Abbreviations

CBT: Cognitive behavior-based therapy
CNO: Clozapine N-oxide
CTL: Control
E/I balance: Excitatory and inhibitory balance
FST: Forced swim test
MDD: Major depressive disorder
mPFC: Medial prefrontal cortex
OFT: Open field test
PL: Prelimbic
RS: Restraint stress
TST: Tail suspension test

## References

[1] Wetherall K, Robb KA, O’Connor RC. Social rank theory of depression: a systematic review of self-perceptions of social rank and their relationship with depressive symptoms and suicide risk. J Affect Disorders. 2019;246:300–19. 10.1016/j.jad.2018.12.045 30594043 10.1016/j.jad.2018.12.045

[2] Langner CA, Epel ES, Matthews KA, Moskowitz JT, Adler NE. Social hierarchy and depression: the role of emotion suppression. J Psychol. 2012;146:417–36. 10.1080/00223980.2011.652234 22808688 10.1080/00223980.2011.652234 PMC5357557

[3] Larrieu T, Sandi C. Stress-induced depression: is social rank a predictive risk factor? Bioessays. 2018;40. 10.1002/bies.201800012 ARTN 1800012. 10.1002/bies.201800012 29869396

[4] Wang J, Geng LN. Effects of socioeconomic status on physical and psychological health: lifestyle as a mediator. Int J Env Res Pub He. 2019;16. 10.3390/ijerph16020281 ARTN 281. 10.3390/ijerph16020281 PMC6352250 30669511

[5] Johnson SL, Leedom LJ, Muhtadie L. The dominance behavioral system and psychopathology: evidence from self-report, observational, and biological studies. Psychol Bull. 2012;138:692–743. 10.1037/a0027503 22506751 10.1037/a0027503 PMC3383914

[6] Park MJ, Seo BA, Lee B, Shin HS, Kang MG. Stress-induced changes in social dominance are scaled by AMPA-type glutamate receptor phosphorylation in the medial prefrontal cortex. Sci Rep. 2018;8:15008. 10.1038/s41598-018-33410-1 30301947 10.1038/s41598-018-33410-1 PMC6177388

[7] Fan Z et al. Neural mechanism underlying depressive-like state associated with social status loss. Cell. 2023;186:560–576 e517. 10.1016/j.cell.2022.12.033 10.1016/j.cell.2022.12.033 36693374

[8] Edson TC et al. A large-scale prospective study of big wins and their relationship with future involvement and risk among actual online sports bettors. Comput Hum Behav. 2023;142. 10.1016/j.chb.2023.107657 ARTN 107657.

[9] Zhou TT, et al. History of winning remodels thalamo-PFC circuit to reinforce social dominance. Science. 2017;357:162–+. 10.1126/science.aak9726 28706064 10.1126/science.aak9726

[10] Page L, Coates J. Winner and loser effects in human competitions. Evidence from equally matched tennis players. Evol Hum Behav. 2017;38:530–5. 10.1016/j.evolhumbehav.2017.02.003 10.1016/j.evolhumbehav.2017.02.003

[11] Fuxjager MJ, Marler CA. How and why the winner effect forms: influences of contest environment and species differences. Behav Ecol. 2010;21:37–45. 10.1093/beheco/arp148 10.1093/beheco/arp148

[12] Beaugrand JP, Payette D, Goulet C. Conflict outcome in male green swordtail fish dyads (Xiphophorus helleri): interaction of body size, prior dominance/subordination experience, and prior residency. Behaviour. 1996;133:303–19. 10.1163/156853996x00161 10.1163/156853996x00161

[13] Hanton S, Mellalieu SD, Hall R. Self-confidence and anxiety interpretation: a qualitative investigation. Psychol Sport Exerc. 2004;5:477–95. 10.1016/S1469-0292(03)00040-2 10.1016/S1469-0292(03)00040-2

[14] Callan MJ, Kay AC, Dawtry RJ. Making sense of misfortune: deservingness, self-esteem, and patterns of self-defeat. J Pers Soc Psychol. 2014;107:142–62. 10.1037/a0036640 24956317 10.1037/a0036640 PMC4076324

[15] Noh K, et al. Cortical astrocytes modulate dominance behavior in male mice by regulating synaptic excitatory and inhibitory balance (26, Pg 1541, 2023). Nat Neurosci. 2023;26:2250–2250. 10.1038/s41593-023-01470-w 37563296 10.1038/s41593-023-01470-w

[16] Codeluppi SA, et al. Prefrontal cortex astroglia modulate anhedonia-like behavior. Mol Psychiatr. 2023;28:4632–41. 10.1038/s41380-023-02246-1 10.1038/s41380-023-02246-1 PMC10914619 37696873

[17] Banasr M, Duman RS. Glial loss in the prefrontal cortex is sufficient to induce depressive-like behaviors. Biol Psychiat. 2008;64:863–70. 10.1016/j.biopsych.2008.06.008 18639237 10.1016/j.biopsych.2008.06.008 PMC2709733

[18] Banasr M, et al. Glial pathology in an animal model of depression: reversal of stress-induced cellular, metabolic and behavioral deficits by the glutamate-modulating drug riluzole. Mol Psychiatr. 2010;15:501–11. 10.1038/mp.2008.106 10.1038/mp.2008.106 PMC3347761 18825147

[19] Miguel-Hidalgo JJ, et al. Glial fibrillary acidic protein immunoreactivity in the prefrontal cortex distinguishes younger from older adults in major depressive disorder. Biol Psychiat. 2000;48:861–73. 10.1016/S0006-3223(00)00999-9 11063981 10.1016/S0006-3223(00)00999-9

[20] Öngür D, Drevets WC, Price JL. Glial reduction in the subgenual prefrontal cortex in mood disorders. P Natl Acad Sci USA. 1998;95:13290–5. 10.1073/pnas.95.22.13290 10.1073/pnas.95.22.13290 PMC23786 9789081

[21] Si XH, Miguel-Hidalgo JJ, O’Dwyer G, Stockmeier CA, Rajkowska G. Age-dependent reductions in the level of glial fibrillary acidic protein in the prefrontal cortex in major depression. Neuropsychopharmacol. 2004;29:2088–96. 10.1038/sj.npp.1300525 10.1038/sj.npp.1300525 PMC3146059 15238995

[22] Steinley D. -means clustering:: a half-century synthesis. Brit J Math Stat Psy. 2006;59:1–34. 10.1348/000711005x48266 10.1348/000711005x48266 16709277

[23] Steinley D. Profiling local optima in K-means clustering: developing a diagnostic technique. Psychol Methods. 2006;11:178–92. 10.1037/1082-989x.11.2.178 16784337 10.1037/1082-989x.11.2.178

[24] Dugatkin LA. Winner and loser effects and the structure of dominance hierarchies. Behav Ecol. 1997;8:583–7. 10.1093/beheco/8.6.583 10.1093/beheco/8.6.583

[25] Hsu YY, Wolf LL. The winner and loser effect: integrating multiple experiences. Anim Behav. 1999;57:903–10. 10.1006/anbe.1998.1049 10202098 10.1006/anbe.1998.1049

[26] Ng TH, Alloy LB, Smith DV. Meta-analysis of reward processing in major depressive disorder reveals distinct abnormalities within the reward circuit. Transl Psychiat. 2019;9. 10.1038/s41398-019-0644-x ARTN 293. 10.1038/s41398-019-0644-x PMC6848107 31712555

[27] Geugies H, et al. Impaired reward-related learning signals in remitted unmedicated patients with recurrent depression. Brain. 2019;142:2510–22. 10.1093/brain/awz167 31280309 10.1093/brain/awz167 PMC6734943

[28] Heshmati M, Russo SJ. Anhedonia and the brain reward circuitry in depression. Curr Behav Neurosci Rep. 2015;2:146–53. 10.1007/s40473-015-0044-3 26525751 10.1007/s40473-015-0044-3 PMC4626008

[29] Admon R, Pizzagalli DA. Dysfunctional reward processing in depression. Curr Opin Psychol. 2015;4:114–8. 10.1016/j.copsyc.2014.12.011 26258159 10.1016/j.copsyc.2014.12.011 PMC4525714

[30] Gabbay V, et al. Striatum-based circuitry of adolescent depression and anhedonia. J Am Acad Child Psy. 2013;52:628–41. 10.1016/j.jaac.2013.04.003 10.1016/j.jaac.2013.04.003 PMC3762469 23702452

[31] Ding YD et al. Reduced nucleus accumbens functional connectivity in reward network and default mode network in patients with recurrent major depressive disorder. Transl Psychiat. 2022;12. 10.1038/s41398-022-01995-x ARTN 236. 10.1038/s41398-022-01995-x PMC9170720 35668086

[32] Hu YQ, Zhao CQ, Zhao HF, Qiao J. Abnormal functional connectivity of the nucleus accumbens subregions mediates the association between anhedonia and major depressive disorder. Bmc Psychiatry. 2023;23. 10.1186/s12888-023-04693-0 ARTN 282. 10.1186/s12888-023-04693-0 PMC10122393 37085792

[33] Bagot RC et al. Ventral hippocampal afferents to the nucleus accumbens regulate susceptibility to depression (vol 6, 7062, 2015). Nat Commun. 2015;6. 10.1038/ncomms8626 ARTN 7626. 10.1038/ncomms8062 PMC4430111 25952660

[34] Kim S, Kwon J, Park MG, Lee CJ. Dopamine-induced astrocytic Ca signaling in mPFC is mediated by MAO-B in young mice, but by dopamine receptors in adult mice. Molecular Brain. 2022;15. 10.1186/s13041-022-00977-w ARTN 90. 10.1186/s13041-022-00977-w PMC9670619 36397051

[35] Pittolo S et al. Dopamine activates astrocytes in prefrontal cortex via receptors. Cell Reports. 2022;40. 10.1016/j.celrep.2022.111426 ARTN 111426. 10.1016/j.celrep.2022.111426 PMC9555850 36170823

[36] Driessen E, Hollon SD. Cognitive behavioral therapy for mood disorders: efficacy, moderators and mediators. Psychiat Clin N Am. 2010;33:537–+. 10.1016/j.psc.2010.04.005 10.1016/j.psc.2010.04.005 PMC2933381 20599132

[37] Passmore TA, Lewy AJ. Practice guideline for the treatment of patients with major depressive disorder, 2nd edition. J Clin Psychiat. 2002;63:371–371. 10.4088/JCP.v63n0416a

[38] Ferguson JMSSRI, Antidepressant Medications. Adverse effects and tolerability. Prim Care Companion J Clin Psychiatry. 2001;3:22–7. 10.4088/pcc.v03n0105 15014625 10.4088/pcc.v03n0105 PMC181155

[39] Ungvari Z, Tarantini S, Yabluchanskiy A, Csiszar A. Potential adverse cardiovascular effects of treatment with fluoxetine and other selective serotonin reuptake inhibitors (SSRIs) in patients with geriatric depression: implications for atherogenesis and cerebromicrovascular dysregulation. Front Genet. 2019;10. 10.3389/fgene.2019.00898 ARTN 898. 10.3389/fgene.2019.00898 PMC6764114 31616477

[40] Botia JA et al. An additional k-means clustering step improves the biological features of WGCNA gene co-expression networks. Bmc Systems Biology. 2017;11. 10.1186/s12918-017-0420-6 ARTN 47. 10.1186/s12918-017-0420-6 PMC5389000 28403906

[41] Yaghouby F, Sunderam S, SegWay. A simple framework for unsupervised sleep segmentation in experimental EEG recordings. Methodsx. 2016;3:144–55. 10.1016/j.mex.2016.02.003 27014592 10.1016/j.mex.2016.02.003 PMC4792881

[42] Dombeck DA, Graziano MS, Tank DW. Functional clustering of neurons in motor cortex determined by cellular resolution imaging in awake behaving mice. J Neurosci. 2009;29:13751–60. 10.1523/JNEUROSCI.2985-09.2009 19889987 10.1523/JNEUROSCI.2985-09.2009 PMC2872549

[43] Tseng GC. Penalized and weighted K-means for clustering with scattered objects and prior information in high-throughput biological data. Bioinformatics. 2007;23:2247–55. 10.1093/bioinformatics/btm320 17597097 10.1093/bioinformatics/btm320

